# Exploring the cross-sectional association between outdoor recreational facilities and leisure-time physical activity: the role of usage and residential self-selection

**DOI:** 10.1186/s12966-018-0689-x

**Published:** 2018-06-18

**Authors:** Joreintje D. Mackenbach, Maria G. Matias de Pinho, Eline Faber, Nicole den Braver, Rosa de Groot, Helene Charreire, Jean-Michel Oppert, Helga Bardos, Harry Rutter, Sofie Compernolle, Ilse De Bourdeaudhuij, Jeroen Lakerveld

**Affiliations:** 1 Department of Epidemiology and Biostatistics, Amsterdam Public Health Research Institute, VU University Medical Center, De Boelelaan 1089b, 1081HV, Amsterdam, the Netherlands; 2 Department of Donorstudies, Sanquin Research, Amsterdam, the Netherlands; 3 Equipe de Recherche en Epidémiologie Nutritionnelle (EREN), Centre de Recherche en Epidémiologie et Statistiques, Inserm (U1153), Inra (U1125), Cnam, Université Paris 13, Bobigny, France; 4 Sorbonne Universités, Université Pierre et Marie Curie, Université Paris 06; Institute of Cardiometabolism and Nutrition (ICAN); Department of Nutrition, Pitié-Salpêtrière Hospital, Assistance Publique-Hôpitaux de Paris (AP-HP), Paris, France; 5 Department of Preventive Medicine, Faculty of Public Health, University of Debrecen, Debrecen, Hungary; 6 Centre for Global Chronic Conditions, London School of Hygiene and Tropical Medicine, London, England; 7 Department of Movement and Sport Sciences, Ghent University, Watersportlaan 2, 9000 Ghent, Belgium

**Keywords:** Built environment, Motivations, Multilevel analysis, Perceptions, Selection effects, Usage

## Abstract

**Background:**

The availability of outdoor recreational facilities is associated with increased leisure-time physical activity (PA). We investigated how much of this association is attributable to selection effects, and explored whether usage of recreational facilities was an explanatory mechanism.

**Methods:**

We analysed data from 5199 participants in the SPOTLIGHT survey residing in five European urban regions. Adults completed a survey and a Google Street View-based virtual audit was conducted to objectively measure the availability of outdoor recreational facilities in the residential neighbourhood. We used negative binomial GEE models to examine the association between objective and subjective availability of outdoor recreational facilities and leisure-time PA, and explored whether this association was attenuated after adjustment for socioeconomic status and preference for neighbourhoods with recreational facilities (as indicators of self-selection). We examined whether reported use of recreational facilities was associated with leisure-time PA (as explanatory mechanism), and summarized the most important motivations for (not) using recreational facilities.

**Results:**

Subjective – but not objective – availability of outdoor recreational facilities was associated with higher levels of total leisure-time PA. After adjustment for self-selection (which attenuated the association by 25%), we found a 25% difference in weekly minutes of total leisure-time PA between individuals with and without self-reported availability of outdoor recreational facilities. For our study population, this translates to about 28 min per week. Participants who reported outdoor recreational facilities to be present but indicated not to use them (RR = 1.19, 95% CI = 1.03;1.22), and those reporting outdoor recreational facilities to be present and to use them (RR = 1.33, 95% CI = 1.22, 1.45) had higher levels of total leisure-time PA than those who reported outdoor recreational facilities not to be present. Proximity to outdoor recreational facilities was the most important motivation for use.

**Conclusion:**

The modest attenuation in the association between availability of outdoor recreational facilities and self-reported leisure-time PA suggests that individuals’ higher activity levels may be due more to the perceived availability of outdoor recreational facilities than to self-selection. The use of these facilities seemed to be an important underlying mechanism, and proximity was the main motivator for using recreational facilities.

**Electronic supplementary material:**

The online version of this article (10.1186/s12966-018-0689-x) contains supplementary material, which is available to authorized users.

## Background

Outdoor recreational facilities such as parks, trail running routes, soccer courts and skate parks provide free opportunities for leisure-time physical activity (PA) and are potentially available to large numbers of individuals. In line with other studies focusing on the association between the built environment and PA [1–5], the availability of parks and other outdoor recreational facilities has been linked to higher levels of PA [4, 6–9]. This may suggest that increasing the availability of outdoor recreational facilities might contribute to the promotion of PA, at least in urban settings.

However, the underlying mechanisms of the association between the presence of outdoor recreational facilities and PA are not well known. Such insights are important for the design of future interventions and to guide policies for urban design to promote public health.

Other than a causal association, it might be that the relation between outdoor recreational facilities and PA reflects an individuals’ residential self-selection, i.e., residents may choose to live in environments that support their activity preferences (direct self-selection bias). For many people, of not most, other factors than the availability of recreational facilities will drive their choice of where to live. Neighbourhoods with low cost housing may also have few recreational facilities, which could perhaps lead to biases in the association between neighbourhood environments and PA (indirect self-selection bias) [10–14]. Potential bias due to residential self-selection has been identified as a key limitation in built environment research [15], as ignoring it may result in an overestimation of the beneficial impact of some characteristics of the built environment on PA [13, 14]. Previous studies have suggested that the bias caused by residential self-selection is limited [4, 16], but the majority of self-selection studies to date have focused on PA for transport – especially walking [11, 12, 17]. In addition, most of these studies have been conducted in North America and Australia, and these results may not be generalizable to residential self-selection effects in the European urban context [11, 12, 17].

If the relation between outdoor recreational facilities and leisure-time PA is not due to selection effects, this would give rise to hypotheses about the explanatory mechanisms underlying any causal effects. Although the provision of recreational facilities may be a necessary condition for behaviour change, it may not be by itself a sufficient condition [18]. In the evaluation of a natural experiment, Panter et al. showed that the effects of changing the built environment on walking and cycling levels was mainly explained by *the use* of the new infrastructure – much more than by changes in cognitions and perceptions relating to the environment [19]. The actual use of recreational facilities may thus be an important explanatory mechanism, and it is therefore of major importance to study why some individuals use neighbourhood facilities and why others do not. In a systematic literature review, McCormack et al. showed that safety, aesthetics, maintenance and proximity were important characteristics of parks that were associated with park use [20]. However, little is known about the motivations residents may have for using or not using outdoor recreational facilities.

In order to gain a better understanding of the ways in which outdoor recreational facilities contribute to leisure-time PA in a European context, we analysed data from the cross-sectional European SPOTLIGHT study to quantify the role of direct and indirect residential self-selection effects and to study the potential explanatory pathways through which the availability of recreational facilities may influence leisure-time PA.

## Methods

### Study design and sampling

This cross-sectional study was part of the European Commission-funded SPOTLIGHT project [21], with data obtained from five urban regions: Ghent and suburbs (Belgium), Paris and inner suburbs (France), Budapest and suburbs (Hungary), the Randstad (including cities of Amsterdam, Rotterdam, The Hague and Utrecht) in the Netherlands, and Greater London (United Kingdom) [22]. Sixty neighbourhoods were randomly sampled according to their level of residential density and socioeconomic status (SES), and four types of neighbourhoods were obtained: high SES/high residential density, low SES/high residential density, high SES/low residential density, low SES/low residential density [22]. A random sample of the neighbourhoods’ adult inhabitants was then invited to participate in the survey between February and September 2014. A total of 6037 individuals were recruited (10.8%, out of 55,893 invited adults). Local ethics committees in each participating country approved the study and all participants gave informed consent. Further descriptions about recruitment of participants, sampling and characteristics of neighbourhoods, are provided elsewhere [22].

### Measures

Participants completed a survey on their socio-demographics, perceived characteristics of their social and physical environment, energy balance-related behaviours, self-rated weight, height and health status, and perceived barriers to healthy behaviours [22]. In addition, the objective physical neighbourhood environment was characterized using a validated virtual audit tool, as previously described [23].

### Dependent variable: Leisure-time physical activity

Questions from the validated self-administered long version of the International Physical Activity Questionnaire (IPAQ) [24] were used to collect data on leisure-time PA in the last seven days. Leisure-time physical activities include walking for leisure (light intensity) and moderate-to-vigorous physical activities like aerobics, running, or cycling. We used three dependent variables to account for the variety of physical activities that can be performed at outdoor recreational facilities: total leisure-time PA, leisure-time walking, and leisure-time moderate-to-vigorous PA, expressed in minutes per week. The IPAQ showed good reliability (Spearman’s correlation coefficients around 0.8) and acceptable criterion validity (median ρ = 0.3) for adults included in a 12-country study [24]. Given the inability of accelerometers to distinguish between domains of physical activity, no information is available with regard to the separate criterion validity of the questions about leisure-time physical activity.

### Independent variable: Availability of outdoor recreational facilities

As previous studies have indicated possible mismatches between objective and subjective measures of neighbourhood facilities [25, 26], we used an objective as well as a subjective measure of availability of outdoor recreational facilities in the neighbourhood.

For the objective assessment of outdoor recreational facilities, we performed a Google Street View-based virtual audit [23]. Outdoor recreational facilities were defined as any man-made or natural outdoor environments where people can exercise, play sports, or recreate in any other way; e.g. parks, soccer courts, outdoor fitness areas or skate parks. Data were collected by trained researchers for 4486 street segments in 59 neighbourhoods (Google Street View data were not available at the time of the virtual audit for one Hungarian neighbourhood) [27]. Availability of outdoor recreational facilities was defined as the percentage of street segments in a neighbourhood with these facilities present. We subsequently classified neighbourhoods into either having at least one outdoor recreational facility available or having no outdoor recreational facilities available, in order to enable comparisons between this objective measure and the subjective measure. As 87.9% of the participants objectively had at least one outdoor recreational facility available in their neighbourhood, we also divided the variable ‘percentage of street segments in a neighbourhood with outdoor recreational facilities present’ into quartiles and performed sensitivity analyses with this new variable.

For the subjective measure, we asked participants whether ‘open recreation areas (such as parks or playing fields)’ were present in their neighbourhood, and if present, whether they had used them in the last month. Answering options were: (1) present and used, (2) present and not used, and (3) not present. We performed analyses with these three categories separately, as well as with a dichotomized variable representing recreational facilities to be ‘present’ (options 1 and 2 combined) or ‘not present’ (option 3), to allow comparison with the objective measure.

### Self-selection variables

We also asked respondents about factors that influenced their decision to live in that neighbourhood. We separately asked respondents about eight factors (e.g. cost of housing, family/friends living nearby) that might have influenced their decision. We used the item ‘*It is close to recreation facilities, parks or sports facilities’* as an indicator for direct self-selection in relation with the research question asked here.

For the indirect self-selection measure, we used education level as a socioeconomic indicator. Education was self-reported in the survey with multiple but differing categories in each country [22]. We combined these categories to classify the education level of participants as either higher (college or university level) or lower (below college level).

### Covariates

Participants reported their birth year, gender and self-rated health, which was measured with a Visual Analogue Scale ranging from 0 (very unhealthy) to 100 (very healthy) [28]. Urban region was also used as a covariate. We tested the effect of adjusting for season or month in which the survey was completed, but since this changed the results little, we decided to present the associations unadjusted for season or month*.*

### Motivations for the (non-)use of outdoor recreational facilities

If participants indicated that recreational facilities were available in their neighbourhood, and they used them, we asked them about the most important motivation for their usage. Seven answering options were available: ‘distance from home’; ‘it is on my route’; ‘price’; ‘my family/friends go here’; ‘I like to go here’; ‘parking’; ‘other’ (only one option could be chosen).

If participants indicated that outdoor recreational facilities were present, but they did not use them, we asked them about the most important motivation for the lack of use. Five answering options were available: ‘too far from home/work/school’; ‘it is not somewhere I would normally go’; ‘price’; ‘parking’; ‘other’.

#### Statistical analyses

After the exclusion of individuals for whom no objectively measured data on the physical neighbourhood environment were available (*N* = 838), a sample of 5199 participants was included in the analyses. Descriptive statistics were used to provide insight into participants’ characteristics. ANOVA, Chi-square, and Mann-Whitney U tests were used to assess if there were statistically significant differences between groups with and without available outdoor recreational facilities (for the subjective and objective measure).

All variables were examined for non-response, with percentages ranging from < 1% (*age*) to 23.7% (*preference for neighbourhoods with recreational facilities*). Multiple imputations were performed, under the assumption that missing values were missing at random (i.e. missing values are dependent on observed data and not on unobserved data) [29]. Thirty imputed datasets were created by Predictive Mean Matching, based on the percentage of missing values.

The dependency of observations within neighbourhoods and countries was evaluated and revealed relevant clustering of individuals within neighbourhoods. Because of the non-normal distribution and high proportion of zeros in the variable leisure-time PA, negative binomial regression analyses were conducted using generalised estimating equations (GEE) with an exchangeable structure [30] and having the neighbourhood level as grouping variable. The coefficients and 95% confidence intervals (CI) generated from the multivariable GEE negative binomial regression analysis were exponentiated to represent rate ratios and their respective CIs. Rate ratios can be translated into the difference in minutes of leisure-time PA per week between those with and without outdoor recreational facilities available by multiplying the rate ratio with the median leisure-time PA of the reference category.

Age, gender, self-rated health and urban region were first tested as effect modifiers by adding interaction terms to the model. Since none of them were significant (*p* < .10) effect modifiers, they were added to the model as confounders.

We assessed the association between subjective and objective availability of outdoor recreational facilities with total leisure-time PA, adjusted for confounders (Model 1). In Model 2, we added the indirect self-selection variable *education*. In Model 3, we replaced the indirect self-selection variable by the direct self-selection variable *preference for neighbourhoods with recreational facilities*. In Model 4, both self-selection variables were added. To quantify the contribution of self-selection variables to the association between the availability of outdoor recreational facilities and leisure-time PA, we calculated the percentage change in coefficient between Models 2,3 and 4 with Model 1.

To examine if use of recreational facilities was more strongly associated with leisure-time PA than (perceived) availability alone, we assessed the association between perceived availability *and use* of recreational facilities in the neighbourhood. In a last step, we described the most common motivations for (not) using recreational facilities in the neighbourhood using pie charts.

In a sensitivity analysis, we used only complete cases to ensure robustness of findings. These results were comparable to the analyses with imputed data (see Additional file 1: Table S1). In addition, we repeated the analyses with a dichotomized measure of the objective availability of outdoor recreational facilities with quartiles of availability of outdoor recreational facilities, in relation to all three leisure-time PA measures (see Additional file 1: Table S2). Analyses were performed using IBM SPSS statistics for Windows V.23.0. *P* < 0.05 was considered statistically significant.

## Results

Table 1 shows the descriptive statistics for the analytical sample (*N* = 5199). The mean age was 52.2 years (*SD*: 16.3), with 55.3% of the participants being female, and 54.1% more highly educated. The median reported time spent on leisure-time PA was 180 min per week. For most participants, outdoor recreational facilities were objectively available in their neighbourhood of residence (87.9%). Similarly, 88.7% of participants perceived outdoor recreational facilities to be available in their neighbourhood and 61.1% of the participants indicated that their neighbourhood choice was related to the availability of recreational facilities.

**Table 1:** Characteristics of the study population

		Perceived availability of outdoor recreational facilities			Objective availability of outdoor recreational facilities		
	Total (*N* = 5199)	Not available (*N* = 588, 11.3%)	Available (≥1) (*N* = 4616, 88.7%)	*p*-value^a^	Not available (*N* = 631, 12.1%)	Available (*N* = 4573, 87.9%)	*p*-value^a^
Age (years)	52.2 (16.3)	54.6 (16.6)	51.9 (16.3)	< 0.001	51.9 (16)	52.3 (16.4)	0.581
Gender, % women	2851 (55.3%)	299 (51.1%)	2552 (55.9%)	0.030	341 (54.6%)	2510 (55.4%)	0.720
Urban regions (country)				< 0.001			< 0.001
Ghent region (Belgium)	1692 (32.5%)	289 (49.1%)	1403 (30.4%)		374 (59.3%)	1318 (28.8%)	
Paris region (France)	707 (13.6%)	75 (12.8%)	632 (13.7%)		257 (40.7%)	450 (9.8%)	
Greater Budapest (Hungary)	712 (13.7%)	67 (11.4%)	645 (14%)		–	712 (15.6%)	
Randstad region (The Netherlands)	1568 (30.1%)	125 (21.3%)	1443 (31.3%)		–	1568 (34.3%)	
Greater London (UK)	525 (10.1%)	32 (5.4%)	493 (10.7%)		–	525 (11.5%)	
Leisure-time PA^b^ (minutes per week)	180 (60–360)	110 (0–300)	180 (60–375)	< 0.001	120 (20–300)	180 (50–370)	< 0.001
Leisure-time moderate-to-vigorous PA (minutes per week)	40 (0–180)	0 (0–120)	40 (0–180)	< 0.001	0 (0–120)	40 (0–180)	< 0.001
Leisure-time walking (minutes per week)	60 (0–180)	30 (0–120)	60 (0–210)	< 0.001	40 (0–150)	60 (0–210)	< 0.001
Education^c^, % higher	2547 (54.1%)	249 (45.7%)	2298 (55.2%)	< 0.001	341 (60.6%)	2206 (53.2%)	0.001
Preference for neighbourhoods with recreational facilities present^c^, % yes	2428 (61.1%)	128 (27.6%)	2300 (65.6%)	< 0.001	229 (47.9%)	2199 (63%)	< 0.001
Objective availability of outdoor recreational facility, % available	4573 (87.9%)	458 (77.9%)	4115 (89.1%)	< 0.001	NA	NA	–
Perceived availability of outdoor recreational facilities, % available	4616 (88.7%)	NA	NA	–	501 (79.4%)	4115 (90%)	< 0.001

Note: values are means (*SD*), frequencies (%), or median (25th percentile – 75th percentile). *SD* = standard deviation. NA = not applicable. *N* in subgroups may vary due to missing values

^a^*p*-values from Chi-squared tests for nominal variables, from ANOVA tests for normally distributed continuous variables, and from Mann-Whitney U Tests for non-normally distributed continuous and ordinal variables

^b^PA = physical activity. ^c^ Self-selection variables

There were significant differences between participants who reported recreational facilities to be available in their neighbourhood and those who did not. Those with higher perceived availability were more physically active during leisure time, had a higher education and more often responded that the availability of recreational facilities influenced the decision to live in their neighbourhood.

Similarly, we observed significant differences between those who objectively had outdoor recreational facilities available and those who did not. Those having recreational facilities available were more active during leisure time and were more likely to have a preference for neighbourhoods with recreational facilities. In addition, they tended to have a lower educational level.

Table 2 shows the multivariable associations between the objective and perceived availability of outdoor recreational facilities and leisure-time PA in weekly minutes, as well as the magnitude of self-selection bias.

**Table 2:** Availability of outdoor recreational facilities and leisure-time physical activity in weekly minutes (*N* = 5199)

	Total leisure-time physical activity			
	Model 1	Model 2	Model 3	Model 4
	*RR* (95% CI)	*RR* (95% CI)	*RR* (95% CI)	*RR* (95% CI)
Outdoor recreational facilities, self-reported:				
Not available	Ref	Ref	Ref	Ref
Available	**1.32 (1.17; 1.48)**	**1.32 (1.18; 1.48**	**1.24 (1.11; 1.36)**	**1.25 (1.11; 1.40)**
Outdoor recreational facilities, objectively measured:				
Not available	Ref	Ref	Ref	Ref
Available	1.08 (0.97; 1.23)	1.08 (0.96; 1.22)	1.06 (0.94; 1.20)	1.06 (0.94; 1.19)
	**Leisure-time walking**			
	**Model 1**	**Model 2**	**Model 3**	**Model 4**
	*RR* (95%CI)	*RR* (95%CI)	*RR* (95%CI)	*RR* (95%CI)
Outdoor recreational facilities, self-reported:				
Not available	Ref	Ref	Ref	Ref
Available	**1.38 (1.19; 1.60)**	**1.39 (1.20; 1.61)**	**1.28 (1.09; 1.48)**	**1.29 (1.11; 1.50)**
Outdoor recreational facilities, objectively measured:				
Not available	Ref	Ref	Ref	Ref
Available	**1.19 (1.03; 1.38)**	**1.18 (1.02; 1.37)**	**1.17 (1.01; 1.36)**	**1.17 (1.01; 1.35)**
	**Leisure-time moderate-to-vigorous physical activity**			
	**Model 1**	**Model 2**	**Model 3**	**Model 4**
	*RR* (95%CI)	*RR* (95%CI)	*RR* (95%CI)	*RR* (95%CI)
Outdoor recreational facilities, self-reported:				
Not available	Ref	Ref	Ref	Ref
Available	**1.27 (1.07; 1.50)**	**1.27 (1.07; 1.50)**	**1.20 (1.01; 1.44)**	**1.20 (1.01; 1.44)**
Outdoor recreational facilities, objectively measured:				
Not available	Ref	Ref	Ref	Ref
Available	0.94 (0.78; 1.13)	0.94 (0.78; 1.13)	0.91 (0.75; 1.10)	0.91 (0.75; 1.10)

Note: *RR* = Rate ratio. Rate ratios and 95% confidence intervals were derived from multivariable GEE negative binomial regression analysis. **Bold** values represent significant associations (two-sided *p* value < 0.05). Model 1 = Model adjusted for age, gender, self-rated health, and urban region. Model 2 = Model 1 additionally adjusted for education. Model 3 = Model 1 additionally adjusted for preference for neighbourhoods with recreational facilities present. Model 4 = Model 1 additionally adjusted for education and preference for neighbourhoods with recreational facilities present

First, individuals who reported the availability of at least one outdoor recreational facility in their neighbourhood performed 30% more total leisure-time PA (RR_model 1_ = 1.32, 95% CI = 1.17; 1.48). This translates to approximately 35 extra minutes of total leisure-time PA per week compared to individuals who reported that outdoor recreational facilities were not available. Adjustment for *education* (Model 2) did not change the coefficient, while adjustment for *preference for neighbourhoods with recreational facilities* changed the coefficient by 25% (Model 3). After adjustment for both residential self-selection variables (Model 4) individuals who reported outdoor recreational facilities to be available in their neighbourhood had 25% higher levels of total leisure-time PA as compared to those who reported no availability (RR = 1.25, 95% CI = 1.11; 1.40). This could roughly be translated to an additional 28 min of total leisure-time PA per week compared to the individuals within this study population who reported no availability of outdoor recreational facilities.

The association of objectively measured availability of outdoor recreational facilities with total leisure-time PA was non-significant. Yet, a comparable pattern of attenuation in the coefficient was observed after adjustment for self-selection variables as with the analyses with the perceived availability of outdoor recreational facilities.

Perceiving recreational facilities to be available was associated with higher levels of leisure-time walking, both before (RR_model 1_ = 1.38, 95% CI = 1.19; 1.60) and after (RR_model 4_ = 1.29, 1.11; 1.50) adjustment for self-selection variables. Objective availability of recreational facilities was also significantly associated with higher levels of leisure-time walking (RR_model 4_ = 1.17, 95% CI = 1.01; 1.35), but these coefficients were barely attenuated by the inclusion of the self-selection variables *education* and *preference for neighbourhoods with recreational facilities*.

Finally, after adjustment for self-selection, significant associations of perceived availability of recreational facilities with leisure-time moderate-to-vigorous PA were also observed (RR_model 4_ = 1.20, 1.01; 1.44). We did not observe significant associations of leisure-time moderate-to-vigorous PA with objective availability of recreational facilities (RR_model 4_ = 0.91 (0.75; 1.10).

Table 3 shows that individuals who perceived outdoor recreational facilities to be available, and who used them, had 33% higher levels of total leisure-time PA (RR_model 4_ = 1.33, *95%CI* = 1.22; 1.45), with the influence of *education* being virtually zero (Model 2), and *preference for neighbourhoods with recreational facilities* attenuating the coefficient by 21% (Model 3). Individuals who perceived outdoor recreational facilities to be available, but reported not using them, had 19% higher levels of total leisure-time PA. This translates to an additional 21 min of total leisure-time PA per week.

**Table 3:** Self-reported availability and use of outdoor recreational facilities with leisure-time physical activity in weekly minutes (*N* = 5199)

	**Total leisure-time physical activity**			
***Self-reported availability and use of outdoor recreational facilities***	**Model 1**	**Model 2**	**Model 3**	**Model 4**
	*RR* (95% CI)	*RR* (95% CI)	*RR* (95% CI)	*RR* (95% CI)
**Not available**	ref	ref	ref	ref
**Available, not used**	**1.13 (1.04; 1.24)**	**1.13 (1.04; 1.23)**	**1.12 (1.03; 1.23)**	**1.19 (1.03; 1.22)**
**Available, used**	**1.39 (1.28; 1.52)**	**1.39 (1.28; 1.51)**	**1.33 (1.22; 1.46)**	**1.33 (1.22; 1.45)**
	**Leisure-time walking**			
	**Model 1**	**Model 2**	**Model 3**	**Model 4**
***Self-reported availability and use of outdoor recreational facilities***	*RR* (95% CI)	*RR* (95% CI)	*RR* (95% CI)	*RR* (95% CI)
**Not available**	ref	ref	ref	ref
**Available, not used**	**1.16 (1.04; 1.29)**	**1.15 (1.03; 1.28)**	**1.14 (1.02; 1.27)**	**1.13 (1.02; 1.26)**
**Available, used**	**1.25 (1.12; 1.40)**	**1.24 (1.11; 1.39)**	**1.18 (1.05; 1.32)**	**1.17 (1.04; 1.31)**
	**Leisure-time moderate-to-vigorous physical activity**			
	**Model 1**	**Model 2**	**Model 3**	**Model 4**
***Self-reported availability and use of outdoor recreational facilities***	*RR* (95% CI)	*RR* (95% CI)	*RR* (95% CI)	*RR* (95% CI)
**Not available**	ref	ref	ref	ref
**Available, not used**	1.14 (0.99; 1.31)	1.13 (0.99; 1.31)	1.14 (0.99; 1.31)	1.14 (0.99; 1.31)
**Available, used**	**1.59 (1.40; 1.79)**	**1.59 (1.40; 1.80)**	**1.55 (1.36; 1.77)**	**1.55 (1.36; 1.77)**

Note: *RR* = Rate ratio. Rate ratios and 95% confidence intervals were derived from multivariable GEE negative binomial regression analysis. **Bold** values represent significant associations (two-sided *p* value < 0.05). Model 1 = Model adjusted for urban region, self-rated general health, age, and gender. Model 2 = Model 1 and additionally adjusted for education. Model 3 = Model 1 and additionally adjusted for preference for neighbourhoods with recreational facilities present. Model 4 = Model 1 and additionally adjusted for education and preference for neighbourhoods with recreational facilities present

Then, individuals who perceived outdoor recreational facilities to be available, and who used them had 17% higher levels of leisure-time walking, while individuals who perceived outdoor recreational facilities to be available, but reported not using them, had 13% higher levels of leisure-time walking.

Finally, individuals who perceived outdoor recreational facilities to be available, and who used them had 55% higher levels of leisure-time moderate-to-vigorous physical activity, while individuals who perceived outdoor recreational facilities to be available, but reported not using them, did not have significantly higher levels of leisure-time moderate-to-vigorous physical activity.

**Fig. 1:**
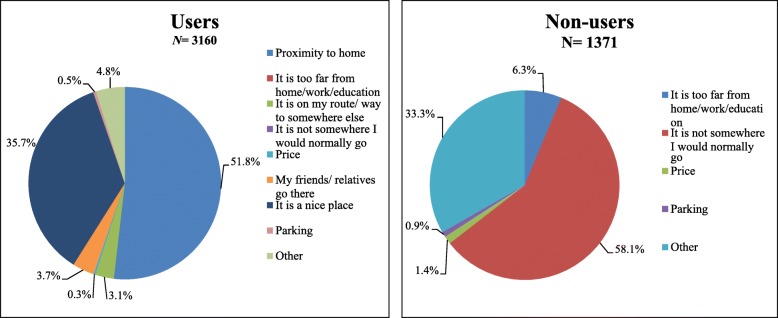
Motivations for using and not using outdoor recreational facilities in the neighbourhood

Figure 1 shows the most important motivations for using and not using recreational facilities in the neighbourhood. ‘Proximity to home’ (51.8%, *N* = 1638) and ‘it is a nice place’ (35.7%, *N* = 1129) were the most frequently reported motivations for using outdoor recreational facilities in the neighbourhood. Least frequently reported motivations were: ‘price’ (0.3%, *N* = 10), and ‘parking’ (0.5%, *N* = 15). The most frequently reported motivation for non-use of outdoor recreational facilities was ‘it is not somewhere I would normally go’ (58.1%, *N* = 797). Within the category ‘other’, ‘personal preferences’ (10.8%, *N* = 10) and ‘no time’ (10.8%, *N* = 10) were most frequently given as the most important other motivation for non-use. Price (1.4%, *N* = 19) and ‘parking’ (0.9%, *N* = 12) did not seem to be important motivators for not using outdoor recreational facilities.

## Discussion

We aimed to obtain a better understanding of how availability of neighbourhood outdoor recreational facilities may contribute to leisure-time PA in a European context, by 1) studying the contribution of direct and indirect self-selection to the association between both perceived and objective availability of outdoor recreational facilities and leisure-time PA in adults, and 2) investigating whether *the use* of outdoor recreational facilities in the neighbourhood may be one mechanism through which they influence leisure-time PA.

As expected and in accordance with previous studies [1–9], perceived availability of outdoor recreational facilities was associated with higher levels of total leisure-time PA, leisure-time walking and moderate-to-vigorous leisure-time PA. Objectively measured availability of outdoor recreational facilities was not associated with total leisure-time PA or leisure-time moderate-to-vigorous PA, but it was associated with higher levels of leisure-time walking. Such a mismatch between perceptions and objective measures has been reported in previous studies [31–33]. Individuals that report outdoor recreational facilities to be available may do so because they use them and thus are more aware of the opportunities for PA [34] – resulting in stronger associations with the perceived measure.

The associations were attenuated after including the self-selection variables in the model, and this was most notable in the models with perceived measures of outdoor recreational facilities. Not adjusting for direct self-selection (i.e., *preference for neighbourhoods with recreational facilities*) resulted in an overestimation of the association between perceived availability of outdoor recreational facilities and total leisure-time PA, as well as associations with leisure-time walking and leisure-time moderate-to-vigorous PA. These results are in accordance with previous studies indicating that self-selection partly explains the association between an activity-friendly environment and PA, even though self-selection measures differ per study [16, 34–36]. Examples of other measures of self-selection include statements such as ‘Having shops and services within walking distance from my home is important to me’, ‘I prefer to bike rather than drive whenever possible’ or ‘To what extent did walkable features play a role in choosing this neighbourhood?’ [37].

We used both a direct and an indirect measure of residential self-selection in this study. Our results suggest that the majority of the self-selection bias was due to the direct self-selection measure, i.e. individuals who preferred neighbourhoods with recreational facilities both resided in neighbourhoods with more facilities, and were more active. Indirect self-selection (with *education* as a socioeconomic indicator) did not play a significant role in these analyses. It may be that our somewhat crude measure of education did not capture residential socio-economic constraints well. Alternatively, it may be that – at least in the areas under study – outdoor recreational facilities in general are in fact equally distributed between more deprived and more affluent neighbourhoods. In that case, having fewer financial resources may not be associated with living in a neighbourhood with fewer recreational facilities. However, we were unable to distinguish between different types of facilities, while these may differ greatly between more deprived and more affluent neighbourhoods [38].

Although there may be other (self-selection) factors that we could not account for in this study, the modest attenuation in the association suggests that individuals’ higher activity levels may be due more to the availability of outdoor recreational facilities than to self-selection. After adjustment for self-selection, we found a 20–25% difference in weekly minutes of leisure-time PA between individuals with and without self-reported availability of outdoor recreational facilities. For our study population, this translates to about 28 min – ~ 20% of the recommended 150 min – per week. There is evidence suggesting that even a relatively modest increase in sustained and regular PA may result in risk reductions of all-cause mortality, and an increase in life expectancy [39]. Given their potential to reach large numbers of individuals over the long term, improvements to neighbourhood environments could lead to major population health benefits even if they only result in relatively small increases in weekly amounts of leisure-time PA at the individual level [7].

Previous studies have shown that the role of self-selection in associations of the built environment with walking is limited [4, 16]. While in our study the association between objectively measured availability of outdoor recreational facilities and leisure-time walking was indeed barely affected by self-selection, this was not the case for the perceived measure of availability of outdoor recreational facilities. The ‘use’ of facilities seems to be an important explanatory mechanism through which outdoor recreational facilities influence leisure-time PA, and this may be different for different types of leisure-time activities. Those who perceived recreational facilities to be available, and used them, had higher levels of leisure-time PA than those perceiving recreational facilities to be available and did not use them, as well as than those perceiving recreational facilities not to be available. For leisure-time moderate-to-vigorous physical activity, the individuals who perceived facilities to be present but did not use them did not have higher levels of physical activity than those who perceived these facilities not to be present in their neighbourhood. The use of such facilities thus seems to be key. The most important motivation for using outdoor recreational facilities in the neighbourhood turned out to be proximity to the facility. Cerin et al. also found that proximity to open spaces, proximity to recreational facilities, and ease of walking partly explained associations between outdoor recreational facilities and leisure-time physical activity [34]. The most important motivation reported by individuals for not using the facilities available in their neighbourhood was that it was a place they would not normally go. This could be explained geographically; i.e. the facility is located in an area they normally do not visit, or it could have a sociological explanation; i.e. the facility is visited by individuals they do not relate to, such as youth hanging around. Alternatively, individuals may simply not be interested in PA. Future (qualitative) studies could further elucidate what factors are most important for the use of facilities.

### Strengths and limitations

Some factors may have limited the results of this study. The use of self-reported data for leisure-time PA –that only allowed for a distinction between walking and other types of PA- could have resulted in over- or underestimation of results [40]. In addition, linking self-reported availability of recreational facilities to self-reported PA may be biased because the measurement error in both reports is correlated (i.e. same-source bias may have arisen). Also, there was discrepancy between the definitions of the objectively and the self-reported measure of outdoor recreational facilities. The self-reported measure comprised open recreation areas (e.g. parks or playing fields) in areas about which participants felt it comprised their neighbourhood, whereas the objective measure comprised outdoor recreational facilities (and as such did not differentiate between open or paid facilities) in administratively defined neighbourhood boundaries. Finally, the low response rate in the survey (11%) may have led to the selective inclusion of healthier and more motivated individuals, therefore the results of this study should be interpreted with caution. Another limitation is that the neighbourhood characteristics only relate to the neighbourhood of residence (as opposed for example to neighbourhood around the place of work where individuals could also perform leisure time PA).

However, this study also benefits from several strengths. We used both objectively measured and self-reported measures of the outdoor recreational facilities, a validated virtual audit tool and a validated measure of PA [23, 24]. External validity was increased by using data from five European countries. In addition, specifically focusing on Europe adds to the literature base, as a large proportion of the existing evidence is based on North American and Australian studies [26]. Also, the large sample of adults, which was recruited from a random sample of neighbourhoods heterogeneous in socio-economic status and residential density, improves external validity.

## Conclusions

We found that perceived - but not objective - availability of outdoor recreational facilities in residents’ neighbourhoods was associated with leisure-time PA. This association was partly due to self-selection; i.e. individuals with higher levels of leisure-time PA selected themselves into certain neighbourhoods because of their preference for neighbourhoods with recreational facilities. However, the role of self-selection in the association was modest, suggesting that the perceived availability of outdoor recreational facilities in a neighbourhood may have a beneficial effect on the level of leisure-time PA. Our study provided a first indication that the use of recreational facilities is an important explanatory mechanism, and that awareness of proximity to facilities is an important motivation for use.

## Additional file

Additional file 1: **Table S1.** Complete case analysis: availability of outdoor recreational facilities with leisure-time physical activity in weekly minutes. **Table S2.** Objectively measured availability of outdoor recreational facilities in quartiles and leisure-time PA (*N* = 5199). (DOCX 26 kb)

## Abbreviations

GEE: generalised estimating eqs.
PA: physical activity
RR: rate ratio
SES: socioeconomic status
