# Supplementary material for: Exploring the cross-sectional association between outdoor recreational facilities and leisure-time physical activity: the role of usage and residential self-selection
Source: Int J Behav Nutr Phys Act. 2018 Jun 18;15:55. doi: 10.1186/s12966-018-0689-x (PMC6006552; doi:10.1186/s12966-018-0689-x)
Supplement: Supplementary file 1 — Table S1. Complete case analysis: availability of outdoor recreational facilities with leisure-time physical activity in weekly minutes. Table S2. Objectively measured availability of outdoor recreational facilities in quartiles and leisure-time PA (N = 5199). (DOCX 26 kb) [file 12966_2018_689_MOESM1_ESM.docx]

**Additional Files**

**Table S1.** Complete case analysis: availability of outdoor recreational facilities with leisure-time physical activity in weekly minutes

|  | **Model 1** | **Model 2** | **Model 3** | **Model 4** |
| --- | --- | --- | --- | --- |
|  | *RR* (95%CI) | *RR* (95%CI) | *RR* (95%CI) | *RR* (95%CI) |
| ***Self-reported availability of outdoor recreational facilities (N= 2971)*** |  |  |  |  |
| **Not available** | ref | ref | ref | ref |
| **Available** | **1.33 (1.16; 1.52)** | **1.31 (1.15; 1.49)** | **1.20 (1.12; 1.34)** | **1.19 (1.02; 1.38)** |
| ***Objective availability of outdoor recreational facilities (N= 3026)*** |  |  |  |  |
| **None** | ref | ref | ref | ref |
| **At least one** | 1.07 (0.94; 1.22) | 1.07 (0.94; 1.22) | 1.01 (0.87; 1.17) | 0.94 (0.86; 1.15) |

Note: *RR* = Rate ratio. Rate ratios and 95% Confidence Intervals were derived from multivariable GEE negative binomial regression analysis. N=3088. **Bold** values represent significant associations (two-sided *p* value < 0.05).
Model 1 = Model adjusted for urban region, self-rated general health, age, education, and gender
Model 2 = Model 1 and additionally adjusted for interest in physical activity
Model 3 = Model 1 and additionally adjusted for preference for neighbourhoods with recreational facilities present
Model 4 = Model 1 and additionally adjusted for combined self-selection variables: interest in physical activity and preference for neighbourhoods with recreational facilities present

**Table S2.** Objectively measured availability of outdoor recreational facilities in quartiles and leisure-time PA (*N*= 5199)

|  |  | ***Total leisure-time PA*** |  |  |
| --- | --- | --- | --- | --- |
| ***Objective availability of outdoor recreational facilities, in quartiles*** | **Model 1** | **Model 2** | **Model 3** | **Model 4** |
|  | *RR* (95%CI) | *RR* (95%CI) | *RR* (95%CI) | *RR* (95%CI) |
| **Q1** | Ref | Ref | Ref | Ref |
| **Q2** | 1.00 (0.90; 1.11) | 1.00 (0.90; 1.11) | 1.00 (0.90; 1.11) | 1.00 (0.90; 1.11) |
| **Q3** | 1.07 (0.97; 1.17) | 1.06 (0.96; 1.17) | 1.08 (0.98; 1.19) | 1.07 (0.98; 1.18) |
| **Q4** | **1.11 (1.01; 1.22)** | **1.11 (1.00; 1.22)** | 1.10 (1.00; 1.21) | **1.10 (1.00; 1.21** |
|  |  | ***Leisure-time walking*** |  |  |
| ***Objective availability of outdoor recreational facilities, in quartiles*** | **Model 1** | **Model 2** | **Model 3** | **Model 4** |
|  | *RR* (95%CI) | *RR* (95%CI) | *RR* (95%CI) | *RR* (95%CI) |
| **Q1** | Ref | Ref | Ref | Ref |
| **Q2** | **1.23 (1.07; 1.41)** | **1.23 (1.08; 1.41)** | **1.23 (1.07; 1.40)** | **1.24 (1.08; 1.42)** |
| **Q3** | **1.22 (1.08; 1.38)** | **1.21 (1.07; 1.36)** | **1.25 (1.10; 1.40)** | **1.23 (1.09; 1.40)** |
| **Q4** | **1.24 (1.09; 1.39)** | **1.24 (1.10; 1.39)** | **1.24 (1.10; 1.40)** | **1.24 (1.10; 1.46)** |
|  |  | ***Leisure-time MVPA*** |  |  |
| ***Objective availability of outdoor recreational facilities, in quartiles*** | **Model 1** | **Model 2** | **Model 3** | **Model 4** |
|  | *RR* (95%CI) | *RR* (95%CI) | *RR* (95%CI) | *RR* (95%CI) |
| **Q1** | Ref | Ref | Ref | Ref |
| **Q2** | **0.80 (0.68; 0.93)** | **0.80 (0.68; 0.93)** | **0.79 (0.68; 0.92)** | **0.79 (0.68; 0.92)** |
| **Q3** | 0.91 (0.79; 1.06) | 0.91 (0.79; 1.06) | 0.91 (0.79; 1.06) | 0.91 (0.79; 1.06) |
| **Q4** | 1.00 (0.86; 1.15) | 0.99 (0.85; 1.16) | 0.99 (0.85; 1.15) | 0.99 (0.85; 1.15) |

Note: *RR* = Rate ratio. *PA* = Physical activity. *MVPA* = Moderate-to-vigorous PA. Rate ratios and 95% Confidence Intervals were derived from multivariable GEE negative binomial regression analysis. **Bold** values represent significant associations (two-sided *p* value < 0.05). Model 1 = Model adjusted for urban region, self-rated general health, age, and gender. Model 2 = Model 1 additionally adjusted for education. Model 3 = Model 1 additionally adjusted for preference for neighbourhoods with recreational facilities present. Model 4 = Model 1 additionally adjusted for education and preference for neighbourhoods with recreational facilities present.
